# Supplementary material for: Empirical methods for controlling false positives and estimating confidence in ChIP-Seq peaks
Source: BMC Bioinformatics. 2008 Dec 5;9:523. doi: 10.1186/1471-2105-9-523 (PMC2628906; doi:10.1186/1471-2105-9-523)
Supplement: Additional File 1 — A variety of html documents from the USeq web site detailing the available applications, their best usage, output file type descriptions, command line menus, etc. [file 1471-2105-9-523-S1.zip › USeqUserGuides/JavaDoc.zip!/JavaDoc/trans/main/T2Parameter.html]

T2Parameter


|  |  |  |
| --- | --- | --- |
| **Overview** \| **Package** \| **Class** \| **Use** \| **Tree** \| **Deprecated** \| **Index** \| **Help** | |  |
| **PREV CLASS**   **NEXT CLASS** | **FRAMES**    **NO FRAMES**     **All Classes** |  |
| SUMMARY: NESTED \| FIELD \| CONSTR \| METHOD | DETAIL: FIELD \| CONSTR \| METHOD |  |


---


## trans.main Class T2Parameter

```
java.lang.Object
  trans.main.T2Parameter
```

---

``` public class T2Parameter extends java.lang.Object ```

Container with info related to runing multiple T2 programs.

---

**Constructor Summary**

`T2Parameter(java.io.File paramFile)`


| **Method Summary** | |
| --- | --- |
| `boolean` | `checkNumberOfReplicas(java.io.File[][] files)` |
| `boolean` | `fetchBoolean(java.lang.String key)` |
| `java.io.File` | `fetchFile(java.lang.String key)` |
| `java.io.File[][]` | `fetchNumberedFiles(java.lang.String prepended, java.io.File directory)` |
| `java.io.File[]` | `fetchNumberedOneFile(java.lang.String prepended, java.io.File directory)` |
| `java.lang.String` | `fetchPossibleValue(java.lang.String key)` |
| `java.lang.String` | `fetchValue(java.lang.String key)` |
| `java.io.File` | `getAppsDirectory()` |
| `java.io.File[][]` | `getControlCelFiles()` |
| `java.io.File` | `getGenomeFastaDirectory()` |
| `java.lang.String` | `getGenomeVersion()` |
| `java.io.File` | `getJava()` |
| `java.io.File` | `getLogFile()` |
| `int` | `getMaxGap()` |
| `int` | `getMaxMemory()` |
| `int` | `getMaxNumberPeaks()` |
| `int` | `getMinimumNumberOfOligos()` |
| `int` | `getMinNumberOligosInSubWin()` |
| `java.io.File[]` | `getNormalizedControlReplicas()` |
| `java.io.File[]` | `getNormalizedTreatmentReplicas()` |
| `java.lang.String` | `getNumberOfIntervals()` |
| `int` | `getNumberOfRandomPermutations()` |
| `int` | `getPeakPickerWindowSize()` |
| `java.lang.String` | `getPermutationType()` |
| `java.io.File` | `getRApp()` |
| `java.io.File[]` | `getRepeatRegionFiles()` |
| `java.io.File` | `getResultsDirectory()` |
| `java.lang.String` | `getResultsName()` |
| `int` | `getSizeOfOligo()` |
| `java.lang.String` | `getStrand()` |
| `int` | `getSubWindowSize()` |
| `java.io.File` | `getSymmetricNullApp()` |
| `double` | `getTargetMedian()` |
| `java.io.File[]` | `getTpmapFiles()` |
| `java.io.File[][]` | `getTreatmentCelFiles()` |
| `int` | `getWindowSize()` |
| `boolean` | `isMakeReducedWindows()` |
| `boolean` | `isMedianScaleUsingControlSeqs()` |
| `boolean` | `isQuantileNormalizeAll()` |
| `boolean` | `isRandomizeData()` |
| `boolean` | `isUsePseudoMedian()` |
| `boolean` | `isUseRandomPermutation()` |
| `boolean` | `isUseSymmetricNull()` |
| `void` | `loadApps()` |
| `void` | `loadGeneral()` |
| `void` | `loadMapsAndRawCelFiles()` |
| `void` | `renameCelFiles()`             Replaces the File pointers to the converted xxx.cela files preserving the input structure. |
| `java.io.File[][]` | `renameToACels(java.io.File[][] f)` |
| `void` | `setNormalizedControlReplicas(java.io.File[] normalizedControlReplicas)` |
| `void` | `setNormalizedTreatmentReplicas(java.io.File[] normalizedTreatmentReplicas)` |

**Methods inherited from class java.lang.Object**

`equals, getClass, hashCode, notify, notifyAll, toString, wait, wait, wait`

**Constructor Detail**

### T2Parameter

```
public T2Parameter(java.io.File paramFile)
```


**Method Detail**

### renameCelFiles

```
public void renameCelFiles()
```

:   Replaces the File pointers to the converted xxx.cela files preserving the input structure.

---


### renameToACels

```
public java.io.File[][] renameToACels(java.io.File[][] f)
```

---


### loadApps

```
public void loadApps()
```

---


### loadGeneral

```
public void loadGeneral()
```

---


### loadMapsAndRawCelFiles

```
public void loadMapsAndRawCelFiles()
```

---


### checkNumberOfReplicas

```
public boolean checkNumberOfReplicas(java.io.File[][] files)
```

---


### fetchNumberedFiles

```
public java.io.File[][] fetchNumberedFiles(java.lang.String prepended,
                                           java.io.File directory)
```

---


### fetchNumberedOneFile

```
public java.io.File[] fetchNumberedOneFile(java.lang.String prepended,
                                           java.io.File directory)
```

---


### fetchValue

```
public java.lang.String fetchValue(java.lang.String key)
```

---


### fetchPossibleValue

```
public java.lang.String fetchPossibleValue(java.lang.String key)
```

---


### fetchFile

```
public java.io.File fetchFile(java.lang.String key)
```

---


### fetchBoolean

```
public boolean fetchBoolean(java.lang.String key)
```

---


### getControlCelFiles

```
public java.io.File[][] getControlCelFiles()
```

---


### getLogFile

```
public java.io.File getLogFile()
```

---


### getResultsDirectory

```
public java.io.File getResultsDirectory()
```

---


### getResultsName

```
public java.lang.String getResultsName()
```

---


### getSizeOfOligo

```
public int getSizeOfOligo()
```

---


### getTpmapFiles

```
public java.io.File[] getTpmapFiles()
```

---


### getTreatmentCelFiles

```
public java.io.File[][] getTreatmentCelFiles()
```

---


### getTargetMedian

```
public double getTargetMedian()
```

---


### getAppsDirectory

```
public java.io.File getAppsDirectory()
```

---


### getMaxMemory

```
public int getMaxMemory()
```

---


### getNormalizedControlReplicas

```
public java.io.File[] getNormalizedControlReplicas()
```

---


### setNormalizedControlReplicas

```
public void setNormalizedControlReplicas(java.io.File[] normalizedControlReplicas)
```

---


### getNormalizedTreatmentReplicas

```
public java.io.File[] getNormalizedTreatmentReplicas()
```

---


### setNormalizedTreatmentReplicas

```
public void setNormalizedTreatmentReplicas(java.io.File[] normalizedTreatmentReplicas)
```

---


### getMinimumNumberOfOligos

```
public int getMinimumNumberOfOligos()
```

---


### getNumberOfRandomPermutations

```
public int getNumberOfRandomPermutations()
```

---


### getRApp

```
public java.io.File getRApp()
```

---


### getSymmetricNullApp

```
public java.io.File getSymmetricNullApp()
```

---


### isUsePseudoMedian

```
public boolean isUsePseudoMedian()
```

---


### isUseRandomPermutation

```
public boolean isUseRandomPermutation()
```

---


### isUseSymmetricNull

```
public boolean isUseSymmetricNull()
```

---


### getWindowSize

```
public int getWindowSize()
```

---


### getGenomeVersion

```
public java.lang.String getGenomeVersion()
```

---


### isMakeReducedWindows

```
public boolean isMakeReducedWindows()
```

---


### getNumberOfIntervals

```
public java.lang.String getNumberOfIntervals()
```

---


### getMaxGap

```
public int getMaxGap()
```

---


### getGenomeFastaDirectory

```
public java.io.File getGenomeFastaDirectory()
```

---


### getMaxNumberPeaks

```
public int getMaxNumberPeaks()
```

---


### getMinNumberOligosInSubWin

```
public int getMinNumberOligosInSubWin()
```

---


### getPeakPickerWindowSize

```
public int getPeakPickerWindowSize()
```

---


### getSubWindowSize

```
public int getSubWindowSize()
```

---


### getRepeatRegionFiles

```
public java.io.File[] getRepeatRegionFiles()
```

---


### getJava

```
public java.io.File getJava()
```

---


### isQuantileNormalizeAll

```
public boolean isQuantileNormalizeAll()
```

---


### isMedianScaleUsingControlSeqs

```
public boolean isMedianScaleUsingControlSeqs()
```

---


### getPermutationType

```
public java.lang.String getPermutationType()
```

---


### isRandomizeData

```
public boolean isRandomizeData()
```

---


### getStrand

```
public java.lang.String getStrand()
```


---


|  |  |  |
| --- | --- | --- |
| **Overview** \| **Package** \| **Class** \| **Use** \| **Tree** \| **Deprecated** \| **Index** \| **Help** | |  |
| **PREV CLASS**   **NEXT CLASS** | **FRAMES**    **NO FRAMES**     **All Classes** |  |
| SUMMARY: NESTED \| FIELD \| CONSTR \| METHOD | DETAIL: FIELD \| CONSTR \| METHOD |  |


---
